# A Pattern Recognition Approach to Acoustic Emission Data Originating from Fatigue of Wind Turbine Blades

**DOI:** 10.3390/s17112507

**Published:** 2017-11-01

**Authors:** Jialin Tang, Slim Soua, Cristinel Mares, Tat-Hean Gan

**Affiliations:** 1 Integrity Management Group, TWI Ltd., Cambridge CB21 6AL, UK; slim.soua@twi.co.uk (S.S.); tat-hean.gan@twi.co.uk (T.-H.G.); 2 College of Engineering, Design and Physical Sciences, Brunel University London, Uxbridge UB8 3PH, UK; cristinel.mares@brunel.ac.uk

**Keywords:** acoustic emission, pattern recognition, fatigue, wind turbine blade, composite, piezoelectric sensors

## Abstract

The identification of particular types of damage in wind turbine blades using acoustic emission (AE) techniques is a significant emerging field. In this work, a 45.7-m turbine blade was subjected to flap-wise fatigue loading for 21 days, during which AE was measured by internally mounted piezoelectric sensors. This paper focuses on using unsupervised pattern recognition methods to characterize different AE activities corresponding to different fracture mechanisms. A sequential feature selection method based on a k-means clustering algorithm is used to achieve a fine classification accuracy. The visualization of clusters in peak frequency−frequency centroid features is used to correlate the clustering results with failure modes. The positions of these clusters in time domain features, average frequency−MARSE, and average frequency−peak amplitude are also presented in this paper (where MARSE represents the Measured Area under Rectified Signal Envelope). The results show that these parameters are representative for the classification of the failure modes.

## 1. Introduction

Success of a wind energy project relies on the reliability of the turbine system. Poor reliability will result in the increase of operation and maintenance costs and the decrease of the wind turbine system lifetime. To improve wind turbine system reliability, it is important to identify critical components and characterize their failure modes, allowing maintenance staff to direct their monitoring, and focus on monitoring methods [1]. Wind turbines suffer from moisture absorption, thermal stress, wind gusts, and lightning strikes. Damage can occur in any part of the turbine–gear box bearings, generator bearings, blades, bolt shears, and load-bearing brace buckles, etc. As blades are the key elements of a wind turbine system and can account for 15–20% of the total system cost, extensive attention has been given to the condition monitoring of blades [2]. Fatigue damage progression represents the main failure factor. To study the contributions of these different types of damage at progressive fatigue stages, a tool with the ability to detect the damage initiation and to monitor failure progress online is needed. Acoustic emission (AE) testing has become a recognized suitable and effective non-destructive technique to investigate and evaluate failure processes in different structural components. The main advantage of AE over other condition monitoring techniques is that detected AE signals can be used to characterize the different damage mechanisms.

The majority of wind turbine blades are made of composite materials, which consist of reinforcing fiber and a matrix. Glass fiber-reinforced polyester or epoxy resin is typically used in wind turbines. Main failure modes in composite materials include fiber failure, matrix cracking, fiber-matrix debonding, and delamination (inter-laminar failure) [3]. The identification of a particular type of damage in composite materials is an emerging research field for AE. Many AE analyses focus on the macroscopic level of time-domain parameter distributions (e.g., peak amplitude and energy) to determine damage initiation and failure mechanisms. Woo, S.C. et al. [4] reviewed the established different amplitude classifications for the damage mechanisms of composites–fiber breakage (i.e., 70 dB–100 dB), fiber/matrix debonding (i.e., 50 dB–70 dB), and matrix cracking (i.e., 30 dB–50 dB), respectively. De Rosa, I.M. et al. [5] summarized previous work to conclude that the AE events of higher energy and frequency can be ascribed to fiber breakages, while events of lower energy and frequency can be ascribed to matrix cracking. Joung-Man Park et al. [6] also concluded that the AE energy released by the fiber breakage is generally larger than that of the matrix cracking and debonding. AE signals can also be well characterized by frequency domain analysis. Qing-Qing Ni et al. [7] concluded that the frequency analysis is an effective way to process AE signals in composite materials. In this regard, the identification of the different damage mechanisms through frequency-based methodologies in AE data analysis from loaded composite materials has also been studied in References [8,9,10], a summary of these results being presented in Table 1. In these experiments, both carbon and glass fiber breakage and fiber pull out showed a relatively high frequency range (above 300 kHz), while the frequency content of matrix cracking corresponded to the low frequency range (30–150 kHz), and delamination and debonding usually occurred in the frequency range in between (200–300 kHz).

The approach of using parameter distribution has the advantage of real-time damage detection, but can also lead to false conclusions due to noise effects in the AE signals. This is especially true for composite materials under the fatigue conditions which are usually present in damage mechanism interactions. Therefore, AE techniques are needed to account for more intricate wave propagation features caused by the anisotropic nature of composite materials and to enable the identification of a large variety of failure modes. It is now possible to detect and capture huge numbers of AE signals, driving a trend for seeking computationally complex algorithms such as pattern recognition to determine the onset of significant AE. For a real structure it is not possible to provide a set of training patterns belonging to multiple damage mechanisms, which thus makes the use of unsupervised pattern recognition techniques more appropriate for these studies.

The first step of developing an unsupervised pattern recognition process is to classify signals into groups based on similarities. This process involves statistical effects, and the key point of successful feature selection to construct fine classification accuracy. In this paper, the presented feature selection method was inspired by D.D. Doan et al. [11], who introduced a sequential method based on the Gustafson-Kessel clustering algorithm. The subset of features is selected by minimizing the Davies-Bouldin (DB) index, which is a metric for the evaluation of classification algorithms. Based on the above methodology, peak frequency and Measured Area under the Rectified Signal Envelope (MARSE) are selected as highly correlated features for the clustering process. The signals are classified into four groups by comparing their features and deciding upon their similarity. The assignment of the clustering results to the fracture mechanisms is achieved by a detailed analysis of the physical meaning of the data [7,8,9,10]. The positions of these clusters in time domain features are also presented, and average frequency, MARSE, and peak amplitude are found to be promising to be useful parameters to represent failure modes.

This paper presents an unsupervised pattern recognition methodology applied to AE signals collected from a long-term fatigue test on a 45.7-m long wind turbine blade. This is the first time that the pattern recognition technique has been applied to a database acquired from such a complex structure in a fatigue testing environment. The applied feature selection algorithm proves to be a powerful tool providing relevant clustering when used together with a k-means algorithm.

## 2. Experiment

### 2.1. Introduction

In service, wind turbines suffer from moisture absorption, thermal stress, wind gusts and lightning strikes. In this paper, we explained that AE signals were collected from a 45.7-m long wind turbine blade loaded in the flapwise direction [12]. During operation, the source of flapwise loading is aerodynamic. The load reaches maximum when the blade position is at 12 o’clock [13]. In preparation for testing, a nominal root bending moment for fatigue loading was selected based on prior experience. The cyclic loading test was performed in three stages over six weeks. During the test, the effective nominal bending moment applied to the blade was increased up to 115% in order to accelerate the damage growth within a short period of time compared with the normal operation time. This introduced a much more challenging environment for AE monitoring due to the high ‘coherent’ noise generated by the movement and friction of the blade. The test environment was optimized to match with the damage level that the blade undergoes in service, where the noise level is much lower.

**Figure 1:**
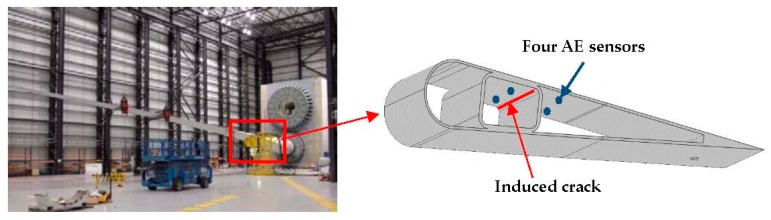
Wind turbine blade under test and acoustic emission (AE) sensors mounted internally on the blade.

AE can be detected in a wide frequency range from 1 kHz to 1 MHz. Based on the reported frequency range of the failure modes, when the structure reaches a critical condition, fiber fracture-generated AE signals generally have frequency increments between 300 kHz and 600 kHz. In this paper, we aim to provide an early warning sign of failure, therefore matrix cracking-, delamination-, and debonding-related AE signals are to be discriminated from noise-generated signals. Studies showed that the frequency content of matrix cracking corresponds to the low frequency range (up to 180 kHz), while delamination and debonding usually have a frequency range in between (200–300 kHz). Thus, the minimal sampling rate needed to avoid aliasing is between 400–600 kHz. Four AE sensors with resonant 150 kHz frequency and a frequency response over the range of 100–450 kHz were used in the experiment. The sensors were connected to an external amplifier with a gain of 34 dB. AE signals were recorded and analyzed using a data acquisition system based on a National Instruments PXIe-1071 card. The sampling rate for this test was 500 kHz to save storage and to improve computational speed. The sensors were mounted internal to the blade, as illustrated in Figure 1. A band pass filter of 5 kHz–250 kHz (Nyquist frequency) was added before the sampler to limit the frequency content of the input filter. AE data acquisition can be affected by numerous factors associated with the electronic instruments, cables, sensors, background noise, and threshold. In this study, to verify the response of our AE system performance, a ‘Hsu pencil source (pencil lead break source)’ was used as the verification source. The calibration followed the ASTM E2374-15 standard. An attenuation test using the pencil lead break method was performed initially on the surface of the blade in order to determine the required number and spacing of the sensors.

The effective loads applied to the blade during the fatigue test were increased in stages to promote crack propagation. A 1 m × 0.05 m × 0.01 m defect was deliberately induced in the region where the AE sensors were located to increase the likelihood of further damage propagation for the purpose of evaluating the proposed AE damage monitoring technique.

### 2.2. Reported Failure Modes

Wind turbines are subject to variable lift force due to gusts, wind shear, up-draught and yaw error, and turbulence. This lifting force causes flapwise vibrations to occur out of the plane of rotation of the blades, whereas edgewise vibrations occur in the plane of rotation [14]. When a wind turbine blade bends in the flapwise direction, the compressed panel produces a downward crushing load normal to the surfaces. The opposite occurs on the lower panel. This nonlinear deformation or ‘flattening’ of the cross-section, known as the Brazier effect, can cause out-of-plane deformation of the spar cap. Since the fibers are mainly aligned along the blade, the spar caps are relatively flexible in the transverse direction. This results in transverse failure, potentially stemming from matrix cracking. In this experiment, delamination and channel cracking initiation growth were observed on the last three days of testing.

## 3. Results

### 3.1. Data Pre-Processing

AE monitoring was carried out throughout all fatigue tests. The detection threshold for the recorded events was set at 40 dB to largely eliminate unwanted noise signals. The threshold for post data processing was increased to 45 dB to decrease the probability of false alarms. Both burst and continuous types of signals were stored for 0.1 s once triggered. For many years, researchers have concentrated on extracting useful information from AE signals by identifying the transient waves in the signal and extracting features. These transient signals, known as AE hits, are commonly determined by a selected detection threshold. AE hit duration is determined by a parameter called Hit Definition Time (HDT), selected as 200 µs in this experiment. Once the hit has been determined, AE hit-based features can be calculated. Time domain features including peak amplitude (A), duration (D), MARSE, rise time (RT), average frequency (AF), as well as frequency domain features including peak frequency (PF) and frequency centroid (FC) are extracted. Signals with fewer than 3 counts and of duration less than 3 µs were regarded as unwanted signals [15]. The original number of hits was 100,855, which was reduced to 74, 852 after the above filter was applied.

**Figure 2:**
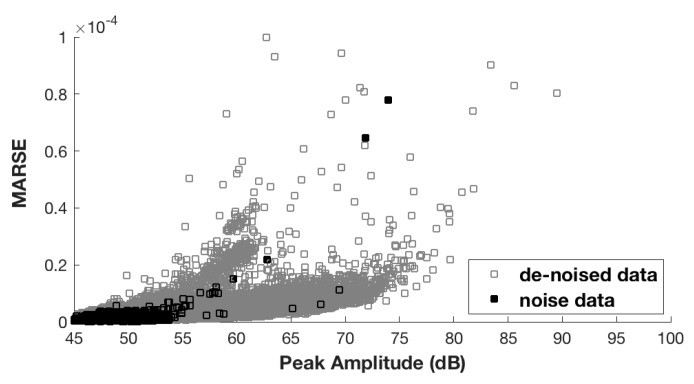
Peak amplitude vs. MARSEdistribution for noise signals and de-noised signals, respectively.

Figure 2 shows the peak amplitude versus MARSE distribution for noise signals and de-noised signals. The noise signals present with low peak amplitude and MARSE values. After feature extraction, feature normalization is an important part of classifier design and of PR in general. In this paper, each parameter is normalized over the range [0, 1] using the equation:

(1) $$X=\left(X-X_{min}\right)/(X_{max}-X_{min})$$

### 3.2. Unsupervised Pattern Recognition

#### 3.2.1. K-Means Clustering

The k-means algorithm is a clustering method that attempts to find a user-specified number of clusters, which are represented by their centroids [16]. It aims at partitioning an *N × n* input dimensional dataset into *c* clusters to minimize the within-cluster sum of squares:

(2) $$\overset{c}{\underset{i=1}{\sum }}\underset{k\in A_{i}}{\sum }{∥x_{k}-v_{i}∥}^{2}$$

where $A_{i}$ is a set of data points in the *i*-th cluster and $v_{i}$ is the mean for those points over cluster *i*. The above equation actually denotes a distance norm. $v_{i}$ is the cluster center:

(3) $$v_{i}=\frac{\sum_{k=1}^{N_{i}}x_{k}}{N_{i}},\;x_{k}\in A_{i}$$

where $N_{i}$ is the number of objectives in $A_{i}$.

K-means clustering has been applied in a wide range of applications due to its high processing speed and simplicity. On the other hand, the requirement for a definite number of clusters as input is often an issue due to the fact that in most tests it is almost impossible to know the number of damage modes in advance.

#### 3.2.2. Feature Selection

In this paper, the principle of feature selection processing is to gradually combine each feature from the available feature space with an initial feature subset. Considering an initial subset of features *S* (empty by default), the algorithm takes each of the available features to update *S*. Feature selection is achieved by minimizing the value of the Davies-Bouldin (DB) index partitioned by the k-means algorithm:

(4) $$DB=\frac{1}{k}\;\overset{k}{\underset{i=1}{\sum }}max_{i\neq j}\left\{\frac{d_{i}+d_{j}}{D_{ij}}\right\}\;\left(i,\;j=1\ldots k\right)$$

where $d_{i}$ is the average Euclidean distance between each point in the ith cluster and the centroid of the ith cluster, and $d_{j}$ is the average Euclidean distance between each point in the jth cluster and the centroid of the jth cluster. $D_{ij}$ denotes the Euclidean distance between the centroids of the ith and jth clusters. The maximum value of $D_{ij}$ represents the worst-case within/between cluster ratio for cluster i. Therefore, the lower the DB value, the better the compactness and the separability within the partition. The computation of the DB index makes use of the Euclidean distance obtained by the k-means algorithm to estimate the distance between AE hits and cluster centers, and finally obtains an estimate of the average within-class distances used in Equation (4) ($d_{i}\;\mathrm{and}\;d_{j}$). An initial feature is chosen for the first interaction, and the feature that minimizes the value of the DB index is then selected. For the next interaction, the initial feature and the selected feature are used together to evaluate by the DB index. The improvement rate IR(k) is defined as:

(5) $$IR\left(k\right)=\frac{DB\left(S_{k}\right)-DB\left(S_{k-1}\right)}{DB\left(S_{k-1}\right)}$$

where $DB\left(S_{k}\right)$ and $DB\left(S_{k-1}\right)$ represent the value of the minimum DB index for the kth and (k−1)th interactions, respectively. A negative value of IR(k) indicates that the DB criterion is improved.

**Figure 3:**
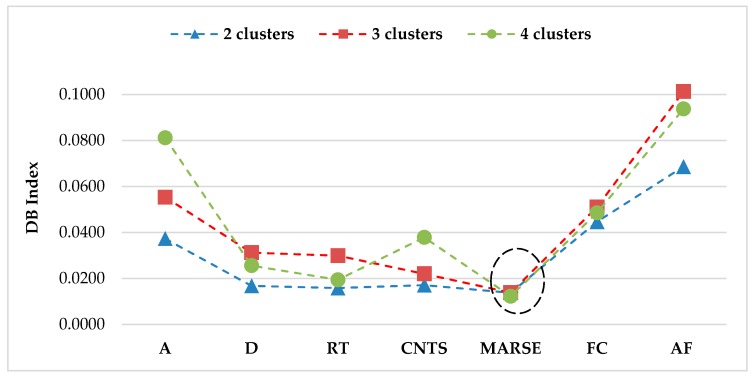
First interaction giving MARSE as the best feature of 2, 3, and 4 clusters.

The selection algorithm was applied with 2, 3, and 4 clusters. Peak frequency has been proven to be an effective parameter to represent different damage mechanisms, and was therefore selected to initiate the feature selection process. At the first interaction, the lowest DB index is given by the combination with MARSE for all three cases, see Figure 3 and Table 2. No more improvement of the DB criterion occurs at the next criterion. Therefore, peak frequency and MARSE are selected for the clustering algorithm.

#### 3.2.3. Optimal Number of Clusters

**Figure 4:**
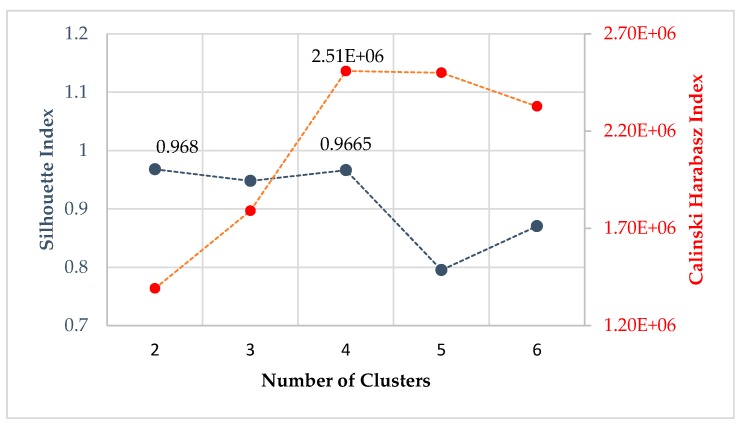
Number of clusters evaluated by the Silhouette index and the Calinski-Harabasz index.

A widely used method to choose an optimal value for the number of clusters is Silhouette analysis. A Silhouette coefficient is defined to study the separation distance between the resulting clusters with a higher value, indicating better cluster quality. A Silhouette value greater than 0.6 generally assures that the clustering is sufficient. R. Gutkin et al. [8] investigated failure in carbon fiber-reinforced plastics using three different pattern recognition techniques including k-means, self-organising maps combined with k-means, and competitive neural networks on AE signals. The number of clusters, k, is chosen between 0 and 2 so that the Silhouette coefficient is maximized. The results from the clustering analysis follow the pattern found in peak frequencies distributions. Li Li et al. [17] identified a framework for the analysis of a link between the damage mode and AE signals originating from the damage initiation and development of 2D and 3D glass/epoxy woven composites loaded in tension. The number of clusters for k-means ++ analysis is evaluated using both Silhouette coefficients and the Davies-Bouldin index. AE signals were divided into four groups which correspond to matrix cracking, fiber/matrix debonding, delamination, and fiber breakage, respectively. Crivelli, D. et al. [18] developed a technique based on Self-Organizing Mapping in conjunction with the k-means algorithm to separate the AE signals from tensile tests of pultruded glass-fiber specimens. The optimal number of clusters is evaluated using three performance indexes including Davies-Bouldin, Silhouette, and Calinski-Harabasz. The result is selected depending on the most voted number of clusters. In this paper, the optimal number of clusters is chosen by taking into account both Calinski-Harabasz and Silhouette quality indexes [19], see Figure 4. The calculation is based on the selected feature database.

The Silhouette index identifies 2 as the best performing clustering number, followed closely by 4, while for the Calinski-Harabasz index the best clustering is 4. Based on the above calculations, 4 is the most voted number of clusters, and is therefore selected as the optimal number of clusters.

#### 3.2.4. Clustering Results

**Figure 5:**
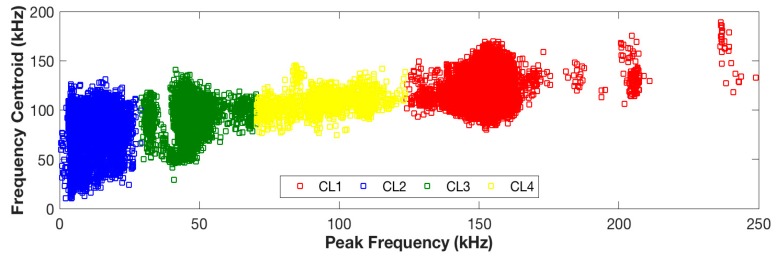
Clustering results: the partition of AE signals on peak frequency–frequency centroid.

Figure 5 shows the partition of AE signals obtained by the above unsupervised pattern recognition method. Frequency features including peak frequency and frequency centroid were used to visualize the position of the signal clusters. It can be observed that the AE signals are well-separated into four clusters in the peak frequency–frequency centroid space. AE signals originating from cluster 1 are related to the high peak frequency. The events with a peak frequency ranging from 0–30 kHz are grouped as cluster 2. Cluster 3 and 4 are identified with a group of signals of peak frequency ranging from 30–70 kHz and 70–120 kHz, respectively.

Table 3 shows the number of events of four clusters based on the clustering results obtained with the selected features on the de-noised database using the k-means clustering algorithm.

#### 3.2.5. AE Source Classification

AE signals are well-separated into four clusters in the peak frequency–frequency centroid space through the above unsupervised pattern recognition methodology. In this section, the characteristic frequency range of each failure mode signals are identified based on the detailed review on the frequency response of the AE signals from composite materials, see Table 1. Based on the clustering results, AE events are classified into four groups of peak frequency ranges from 0–30 kHz, 30–70 kHz, 70–120 kHz, and 120–250 kHz, respectively. The literature review stated that signals generated from matrix cracking and delamination range from 0–150 kHz; therefore, signals from clusters 2, 3, and 4 can be linked with these two fracture mechanisms. As for cluster 1, which corresponds to a higher peak frequency range, signals in this cluster are associated with debonding.

**Figure 6:**
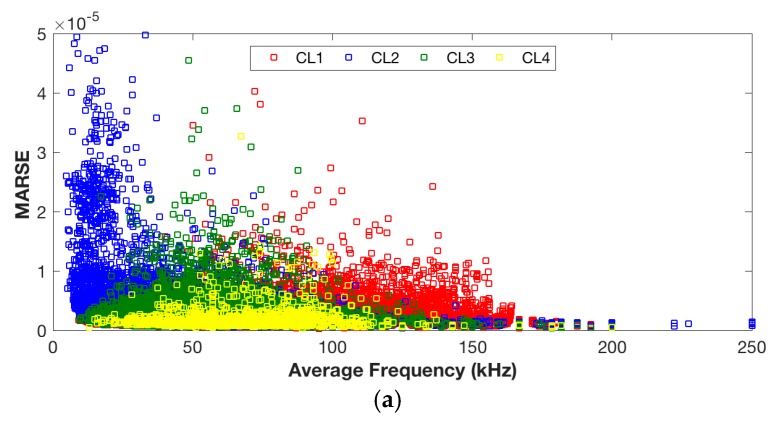

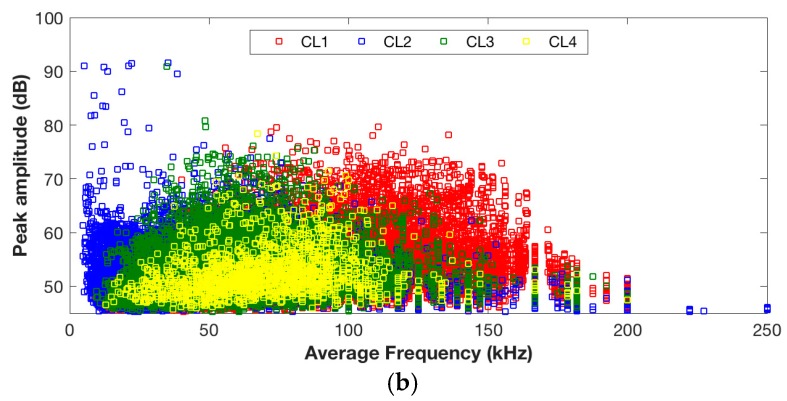
Clustering results: the partition of AE signals on time domain features. (**a**) Clustering results on average frequency vs MARSE; (**b**) Clustering results on average frequency vs. peak amplitude.

The positions of these clusters in time domain features including average frequency, MARSE, and peak amplitude are presented in Figure 6. The objective of analyzing the time domain features was to further discriminate the clusters 2, 3, and 4 into different damage mechanisms by looking into the physical meaning of the signals.

Events in clusters 3 and 4 exhibit a very similar response on time domain features; cluster 2 features a lower average frequency but higher MARSE, see Figure 6a. It is believed that clusters 3 and 4 correspond to the same fracture mechanism and cluster 2 corresponds to a different one. Events in cluster 2 feature a lower peak amplitude (45–60 dB) in comparison with the events in clusters 3 and 4, see Figure 6b. Woo, S. C. et al. [4] concluded that matrix cracking generally has a lower peak amplitude response than delamination. Therefore, in the author’s opinion, events in cluster 2 correspond to matrix cracking, while events in clusters 3 and 4 are generated by delamination.

**Figure 7:**
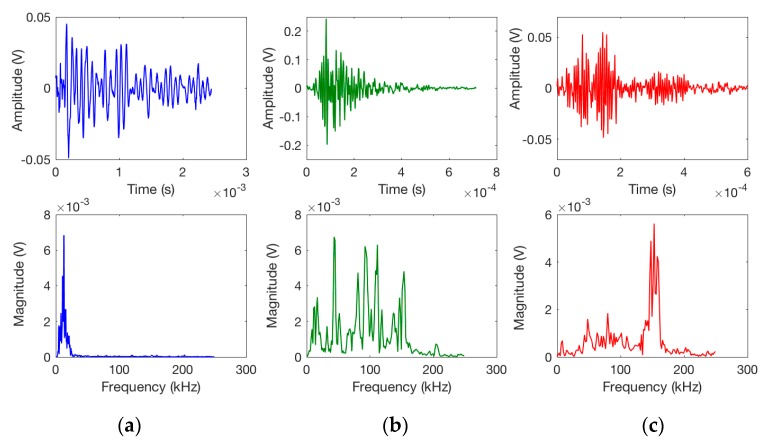
A representative signal in the time domain and frequency domain due to: (**a**) matrix cracking; (**b**) delamination; (**c**) debonding.

A representative signal from each class is shown in Figure 7: the (a) type signal is from cluster 2, with a peak frequency less than 30 kHz, lower peak amplitude, and longer duration (which is the reason for a higher MARSE value); the (b) type signal is related to delamination with a higher peak frequency and higher amplitude, but shorter duration; and the (c) type signal shows a much higher peak frequency and the shortest duration.

**Figure 8:**
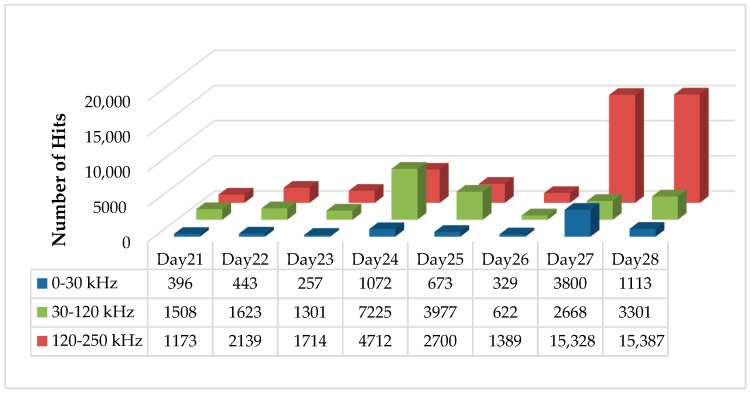
Number of events with peak frequency ranges 0–30 kHz, 30–120 kHz, and 120–250 kHz.

Peak frequency distribution analysis was carried out based on the clustering results. Signals were clustered into three groups with the peak frequency range 0–30 kHz, 30–120 kHz, and 120–250 kHz, respectively, see Figure 8.

It can be observed that various damage mechanisms occur at different speeds, and a large amount of AE activity happened in the peak frequency range from 120 to 250 kHz on days 27 and 28, which is the period when the visual inspection confirmed a severe damage growth.

## 4. Conclusions

This paper presents a pattern recognition methodology applied to AE signals collected from a long-term fatigue test on a 45.7-m long wind turbine blade. Due to the absence of a priori knowledge about the structure, the unsupervised pattern recognition approach was used to classify signals into different damage mechanisms. Through the presented algorithm, the optimal number of clusters and the best features for clustering were specified. The clustering results were assigned to the fracture mechanisms through a detailed analysis of the physical meaning of the data. This part of practice is a great help for future use as a training set for supervised pattern recognition method, which involved a learning process and where each new set of data was processed and classified to one of the previously known and predefined groups.

This is the first time that a pattern recognition technique has been applied to a database acquired from such a complex structure in a fatigue testing environment. The applied feature selection algorithm proved to be a powerful tool to provide good clustering quality when used with a k-means clustering algorithm. The visualization of clusters in peak frequency–frequency centroid feature allowed the correlation of the clustering results with failure modes. This was achieved by adopting the results of a detailed study of the frequency content of AE signals characterizing the failure modes in composite materials. The positions of these clusters in time domain features, average frequency–MARSE, and average frequency–peak amplitude were also investigated in this paper, which promise to be useful parameters to represent failure modes.

In summary, three damage mechanisms have been identified: AE hits with a peak frequency range from 0 to 30 kHz, a relatively higher MARSE, and lower average frequency and peak amplitude values were found to be indicative of matrix cracking. The second group of signals with a peak frequency range from 30 kHz to 120 kHz occurred with a low average frequency and low MARSE signature in the average frequency–MARSE distribution plot, but with a higher peak amplitude. It is believed that this group of signals identifies delamination activities. Signals with a higher peak frequency range and lower MARSE, but with high average frequency values formed a cluster that was considered to indicate higher energetic sources such as debonding. Since the frequency content of AE signals can be altered by the sensor’s frequency response sensitivity, further work should aim at collecting signals from other types of sensors (based on sensor resonances), to further confirm the proposed AE source classification method.

## Figures and Tables

**Table 1:** Frequency analysis results.

Failure Modes	Frequency Range (kHz)			
	Glass/Polyester [9]	Glass/Polypropylene [10]	Carbon/Epoxy [7]	Carbon/Epoxy [8]
Matrix cracking	30–150	×	<100	0–50
Delamination	×	×	×	50–150
Debonding	180–290	100	200–300	200–300
Fiber breakage	300–400	450–550	400–450	400–500
Fiber pull out	180–290	200–300	×	500–600

**Table 2:** DB index values at the first interaction for 2, 3, and 4 clusters.

Features	DB Index		
	2 Clusters	3 Clusters	4 Clusters
Peak Amplitude (A)	0.0373	0.0553	0.0812
Duration (D)	0.0168	0.0312	0.0256
Rise Time (RT)	0.0158	0.0299	0.0194
Counts (CNTS)	0.0171	0.0220	0.0378
MARSE	0.0138	0.0138	0.0122
Frequency Centroid (FC)	0.0446	0.0510	0.0485
Average Frequency (AF)	0.0686	0.1013	0.0937

**Table 3:** Clustering result: the number of events for four clusters.

Cluster	Number of Events
1	44,542
2	8083
3	19,531
4	1577
